# Characterization of Sensitivity of Time Domain MEMS Accelerometer

**DOI:** 10.3390/mi15020227

**Published:** 2024-01-31

**Authors:** Enfu Li, Jiaying Jian, Fan Yang, Zhiyong Ma, Yongcun Hao, Honglong Chang

**Affiliations:** 1 School of Engineering, Huzhou University, Huzhou 313000, China; 02567@zjhu.edu.cn (F.Y.); 02641@zjhu.edu.cn (Z.M.); 2 School of Electronic Information and Engineering, Xi’an Technological University, Xi’an 710021, China; jianjiaying@xatu.edu.cn; 3 Ministry of Education Key Laboratory of Micro and Nano Systems for Aerospace, Northwestern Polytechnical University, Xi’an 710072, China; haoyongcun@nwpu.edu.cn

**Keywords:** MEMS, inertial sensor, accelerometer, time domain measurement, adjustable sensitivity

## Abstract

This paper characterizes the sensitivity of a time domain MEMS accelerometer. The sensitivity is defined by the increment in the measured time interval per gravitational acceleration. Two sensitivities exist, and they can be enhanced by decreasing the amplitude and frequency. The sensitivity with minor nonlinearity is chosen to evaluate the time domain sensor. The experimental results of the developed accelerometer demonstrate that the sensitivities span from −68.91 μs/g to −124.96 μs/g and the 1σ noises span from 8.59 mg to 6.2 mg (amplitude of 626 nm: −68.91 μs/g and 10.21 mg; amplitude of 455 nm: −94.51 μs/g and 7.76 mg; amplitude of 342 nm: −124.96 μs/g and 6.23 mg), which indicates the bigger the amplitude, the smaller the sensitivity and the bigger the 1σ noise. The adjustable sensitivity provides a theoretical foundation for range self-adaption, and all the results can be extended to other time domain inertial sensors, e.g., a gyroscope or an inclinometer.

## 1. Introduction

Microelectromechanical system (MEMS) acceleration sensors have been extensively applied in various fields, including consumer electronics, industrial platforms, infrastructure monitoring, and oil and gas exploration, due to the advantages of their low cost, size, weight, and power (CSWaP) [1,2]. Traditionally, the implementation of acceleration sensors has depended on different transducers. For example, a transducer converts acceleration perturbation into changes in the displacement of a proof mass [3], a transducer converts acceleration perturbation into shifts in the resonant frequency of a resonator [4], or a transducer converts acceleration perturbation into variations in the spatial energy distribution between two coupled resonators [5], according to the literature [6,7].

Based on transducers that convert the applied acceleration perturbation into changes in displacement, the developed accelerometers include capacitive sensors [3,8], tunneling sensors [9], piezoresistive sensors [10], and piezoelectric sensors [11], where both the proof mass and elastic beam of the developed acceleration sensors are under a quasi-static state. The sensitivities of these sensors can be improved via lowering the natural frequency of the sensor. The most straightforward method is to increase the proof mass [12] and use negative electrostatic spring stiffness [13]. A geometric anti-spring system has been chosen to lower the resonant frequency [14]; i.e., anti-springs become softer and their resonant frequency becomes lower with increased input acceleration. Moreover, various methods have also been adopted to increase the sensitivity, including oblique comb electrodes utilizing both the overlapped area and the gap between the movable and stationary electrodes [15], arraying suspended piezoresistive bridges [16], choosing a piezoelectric film with a higher dielectric constant [17], and using parylene as a support diaphragm due to its very low Young’s modulus [18]. Based on transducers that convert the applied acceleration perturbation into resonant frequency shifts or modal shape variations, the currently existing acceleration sensors are resonant sensors [19] and mode-localization sensors [20], respectively, where the proof mass works under a quasi-static state while the elastic beam works under a resonant state. The sensitivity of resonant sensors can be improved by differential resonators [21], multistage leverage mechanisms [22], and flexible resonant beams [19]. And the sensitivity of the mode-localized accelerometer can be improved by lowering the weak coupling coefficient [23]. In all three traditional transducers, the sensitivity should be measured or calibrated before each usage; then, the measured voltage or current represents the acceleration perturbation.

A new transducer that converts the applied acceleration perturbation into changes in time intervals, i.e., a time domain accelerometer, was proposed in theory by the Space and Naval Warfare Systems Center Pacific (SSC Pacific) [24]. For the transducer, the applied acceleration perturbation is solved by measuring varying time intervals between triggering events. A triggering event is generated when a harmonically oscillating mass on a spring goes through the preset displacement reference points (DRPs).

From that moment on, two research groups from the SSC Pacific and Northwestern Polytechnical University (NPU) have been performing studies on time domain sensors, according to the published literature to date. The researchers at the SSC Pacific proposed an implementation method of a time domain device [24] where the DRPs are directly achieved using stacked tunneling electrodes. This time domain sensing mechanism has the potential to achieve a detection limit of ~10^−13^ g in theory [7] because the state-of-the-art time accuracy of 10^−18^ s has already been achieved [25]. Based on the implementation method, the researchers at the SSC Pacific also performed some theoretical work on MEMS time domain inertial sensors including accelerometers and gyroscopes, mainly focusing on the apparatus and method of in-plane inertial devices [26], the intelligent polynomial curve fitting method for inertial devices [27], modeling gyroscope devices [28], and the angular random walk estimation method for gyroscopes [29]. However, none of the time sensors were developed due to the method being hard to implement. As a result, the theoretical results are not experimentally verified.

The researchers at NPU (the authors of this paper) proposed another implementation method of a time domain sensor [30,31], where the output voltage of the capacitance device is used to indirectly represent the displacement of the resonant mass, while the voltage reference points (VRPs) are used to indirectly represent the DRPs. Utilizing a traditional capacitance device and a capacitance-to-voltage (C–V) interface circuit, the indirect representation was achieved. And utilizing this implementation method, a time domain accelerometer was developed, and the time domain sensing method was validated in an experiment. Moreover, we built a virtual accelerometer array using one device based on time domain measurement [31]. Multiple acceleration measurements can be simultaneously performed by the built virtual accelerometer array. The accuracy is improved by combining all the measurements. Furthermore, a feature, the time domain accelerometer’s insensitivity to the changes in the vibration amplitude and the shifts in the resonant frequency, was theoretically found and experimentally validated [7]. With this feature, the time domain sensor has the ability to measure acceleration during the process of attenuation vibration, which has already been validated in an experiment.

However, the sensitivity of time domain inertial sensors has seldom been reported in the published literature to date. And through a comparison of time domain sensors with the three traditional transducer-based accelerometers (Table 1), it can be seen that although the proof mass of the time domain acceleration sensor works under a resonant state, its resonant frequency remains invariable with accelerometer perturbation. Consequently, the sensitivity of the time domain acceleration sensor cannot be evaluated in the same way as that of a resonant sensor: via the increment in the resonant frequency per gravitational acceleration. In addition, although the center point of the oscillation trajectory of the time domain sensor with acceleration perturbation experiences a shift in displacement compared with that of the zero-acceleration oscillation trajectory, and the shifted displacement equals the acceleration, it is hard to chart the displacement of the oscillator and determine its center point [32]. Consequently, the sensitivity of the time domain acceleration sensor cannot be evaluated like that of the transducer because applied acceleration perturbation is converted into displacement changes in proof masses, such as capacitive sensors, piezoresistive sensors, and piezoelectric sensors. Moreover, among the three measured intervals used for solving acceleration, two of them vary with acceleration perturbation [31]. Based on the analysis above, the sensitivity of the time domain accelerometer is thoroughly different from that of the traditional accelerometer. Therefore, in this paper, the characterization of time domain accelerometer sensitivity is presented, including the sensitivity definition, formula, nonlinearity, and adjustable principle.

The rest of this paper is arranged as follows: The sensitivity formula and nonlinearity of the sensitivity as well as the sensitivity adjustment principle are theoretically analyzed in Section 2. In Section 3, the device implementation and experimental methods are described. Experimental verifications and a discussion are presented in Section 4. The final conclusions are summarized in Section 5.

## 2. Characterization of Sensitivity

### 2.1. Definition and Deducing of Sensitivity Formulas

The time domain sensing mechanism is described in the Supporting Information, which is attached for better readability of this paper. The acceleration, which is based on time measurement, is determined by three measured time intervals and can be expressed as a function of these intervals [31].

(1) $$a={\left(\frac{2\pi }{\Delta T}\right)}^{2}\left[\frac{\left(X_{1}-X_{2}\right)}{\cos\left(\pi \frac{\Delta T_{1}}{\Delta T}\right)-\cos\left(\pi \frac{\Delta T_{2}}{\Delta T}\right)}\cos\left(\pi \frac{\Delta T_{1}}{\Delta T}\right)-X_{1}\right]$$

where *X*_1_ and *X*_2_ are predefined DRPs; Δ*T*_1_, Δ*T*_2_, and Δ*T* refer to the measured time intervals. As for the part of Equation (1), $\left(X_{1}-X_{2}\right)/\left[\cos\left(\pi \Delta T_{1}/\Delta T\right)-\cos\left(\pi \Delta T_{2}/\Delta T\right)\right]$ is the measured amplitude *A* of the harmonic oscillation trajectory, while in terms of part of Equation (1), 2π/ΔT is the measured resonant frequency *ω*_0_ of the harmonic oscillation trajectory. To ensure that the measurement range of the time domain accelerometer is symmetrical about zero-acceleration perturbation, DRP *X*_1_ is set equal to zero [31]. When the measured amplitude and the measured resonant frequency are introduced into Equation (1), Equation (1) can be rewritten as

(2) $$a=A\omega_{0}^{2}\cos\left(\omega_{0}\frac{\Delta T_{1}}{2}\right)$$

As seen from Equation (2), the measured acceleration is just determined by varying time interval Δ*T*_1_.

Sensitivity is defined by the change in the measured time interval per one meter, per second squared ($s/m/s^{2}$) or per gravity (s/g, $s/g=9.8\;s/m/s^{2}$). Therefore, the sensitivity is equal to the derivative of the time intervals with respect to acceleration. According to Equation (2), the sensitivity *S*_1_ can be expressed by

(3) $$S_{1}=\frac{d\Delta T_{1}}{da}=\frac{2}{-\omega_{0}^{3}A\sin\left(\omega_{0}\frac{\Delta T_{1}}{2}\right)}\propto \frac{2}{-\omega_{0}^{3}A}$$

**Figure 1:**
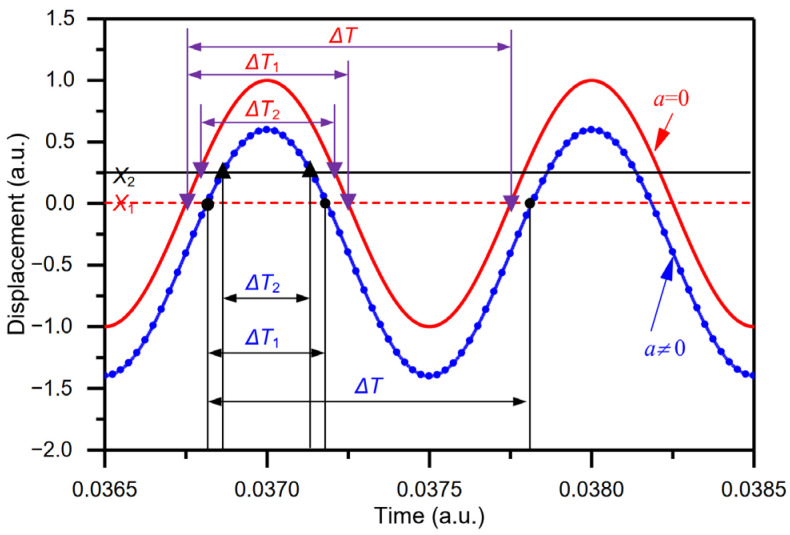
The relationship between time intervals and acceleration perturbation (time intervals Δ*T*_1_ and Δ*T*_2_ vary with acceleration perturbation while time interval Δ*T* is the period of vibration and does not vary with acceleration perturbation).

The time interval Δ*T*_1_ varies with acceleration perturbation, as shown in Figure 1. Similarly, the sensitivity *S*_2_ can be also defined by the increment in the measured time interval Δ*T*_2_ per one meter per second squared and can be expressed by

(4) $$S_{2}=\frac{d\Delta T_{2}}{da}=\frac{2}{-\omega_{0}^{3}A\sin\left(\omega_{0}\frac{\Delta T_{2}}{2}\right)}\propto \frac{2}{-\omega_{0}^{3}A}$$

As shown in Figure 1, the measured time interval Δ*T* is equal to the period of harmonic vibration, while does not vary with the acceleration perturbation and thus cannot be used to define the sensitivity.

### 2.2. Making a Better Choice between Sensitivities S_1_ and S_2_ Based on Nonlinearity for Evaluating Time Domain Sensor

Comparing sensitivity *S*_1_ (Equation (3)) and sensitivity *S*_2_ (Equation (4)), the difference between the two sensitivities *S*_1_ and *S*_2_ lies in the measured time intervals Δ*T*_1_ and Δ*T*_2_. Other than that, the time interval Δ*T*_1_ is determined by the DRP *X*_1_, and the time interval Δ*T*_2_ is confirmed by the DRP *X*_2_ (Figure 1). Moreover, due to the nonlinearity of $\sin\left(\omega_{0}\Delta T_{1}/2\right)$ or $\sin\left(\omega_{0}\Delta T_{2}/2\right)$ in time interval Δ*T*_1_ or Δ*T*_2_, the sensitivities *S*_1_ and *S*_2_ (Equations (3) and (4)) of the time domain accelerometer are nonlinear, as seen for a tilt sensor based on an accelerometer [33]. Furthermore, when the range is symmetric about zero acceleration, the measured time interval Δ*T*_1_ is symmetrical, and so is the angle $\omega_{0}\Delta T_{1}/2$, while the measured time interval Δ*T*_2_ and the angle $\omega_{0}\Delta T_{2}/2$ are not symmetrical. Consequently, the sensitivity *S*_1_ has a smaller nonlinearity than the sensitivity *S*_2_. Thus, the sensitivity *S*_1_ is chosen to evaluate the sensitivity of the time domain acceleration sensor.

**Figure 2:**
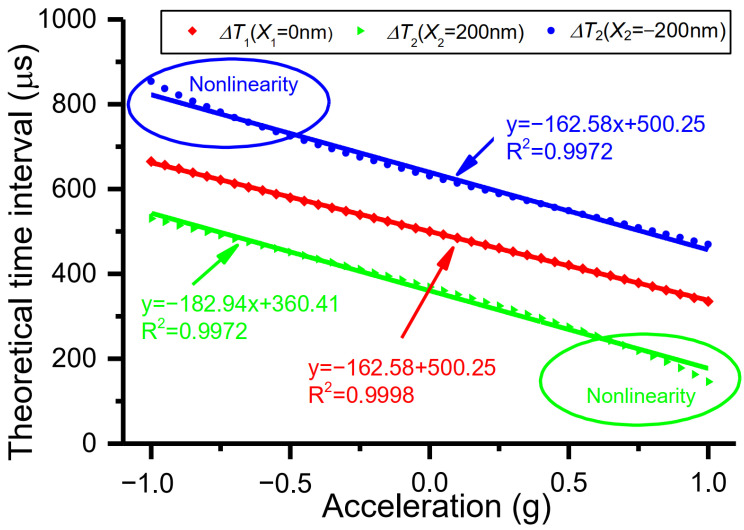
Sensitivities and their nonlinearity of the time domain sensor under different DRPs in the measurement range from −1 g to 1 g.

The sensitivity is simulated according to Figure 1. Discrete data are generated based on the parameters of vibration amplitude, resonant frequency, applied acceleration perturbation, and time clock resolution. Then, the measurement of time intervals is carried out using the method presented in [31], and acceleration is solved. Assuming that the vibration amplitude of 500 nm, the resonant frequency of 1 KHz, and the time clock resolution of 10^−7^ s are preset, the simulated sensitivity *S*_1_ is −162.58 μs/g (*R*^2^ = 0.9998) when *X*_1_ is 0 nm, while the sensitivity *S*_2_ is −182.94 μs/g (*R*^2^ = 0.9972) when *X*_2_ is 200 nm or −200 nm (Figure 2). The simulated sensitivity *S*_1_ is smaller than sensitivity *S*_2_, especially in the situation where the nonlinearity of the sensitivity *S*_1_ is smaller than that of the sensitivity *S*_2_, which is in line with the aforementioned analysis. Therefore, the sensitivity *S*_1_ is a better choice for evaluating the time domain accelerometer.

### 2.3. Adjustment Principle of Sensitivity

It can be seen from Equations (3) and (4) that the sensitivity of the sensor is inversely proportional to the vibration amplitude and the third power of resonant frequency. Comparatively speaking, the resonant frequency has a relatively great influence on the sensitivity. For example, when the vibration amplitude is enlarged by a factor of k, the sensitivity is reduced by a factor of k, whereas when the resonant is enlarged by a factor of k, the sensitivity is reduced by a factor of $k^{3}$.

By adjusting the vibration amplitude and resonant frequency, the sensitivity can be modified. The amplitude can be adjusted by changing the electrostatic driving force, while the resonant frequency can be altered by adjusting the stiffness coefficient of the elastic beam or varying the proof mass. However, once the mechanical structure of the time domain sensor is fabricated, the elastic beam and proof mass cannot be directly modified to change the resonant frequency. Instead, the resonant frequency can be changed by applying a DC bias voltage to introduce electrostatic negative stiffness [13]. This adjustment in resonant frequency allows for the fine-tuning of sensitivity. Furthermore, the performance of the time domain sensor can be tuned based on the adjustable sensitivity. In addition, a time domain sensor with measurement range self-adaption can be theoretically achieved.

## 3. Device Implementation and Experimental Method

**Figure 3:**
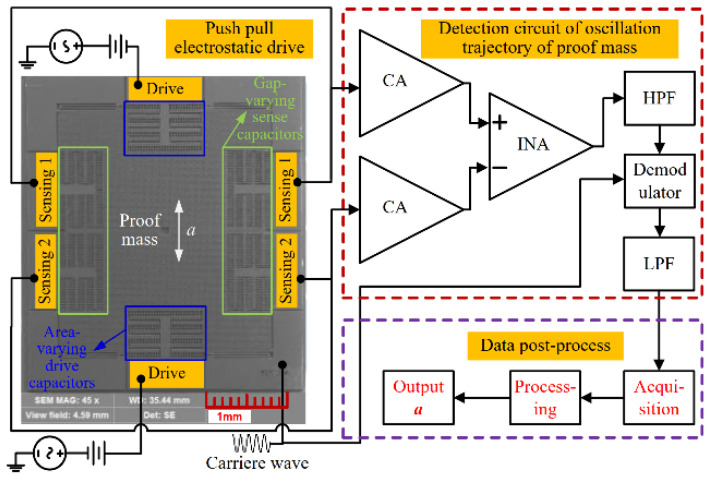
SEM image of developed accelerometer and block diagram of acceleration measurement.

The scanning electron microscope (SEM) image of the developed time domain accelerometer and the block diagram of acceleration measurement are shown in Figure 3. The fabricated device consists of drive capacitors, sense capacitors, and a proof mass on a spring, which converts the changes in displacement caused by acceleration perturbation into changes in capacitance. The detection circuit of the oscillation trajectory transforms the changes in capacitance into those in voltage. Therefore, the displacement caused by acceleration perturbation is represented by the output voltage signal. Correspondingly, the DRPs are denoted by the VRPs. Finally, data sampling is carried out from the output voltage signal utilizing National Instrument serial data acquisition (DAQ) [34]. Sampling data are post-processed through MATLAB 2012b, as displayed in Figure 3. As a result, the time intervals can be obtained, and then the acceleration can be solved from the obtained time intervals. Detailed descriptions have been given in the literature [7,31].

**Figure 4:**
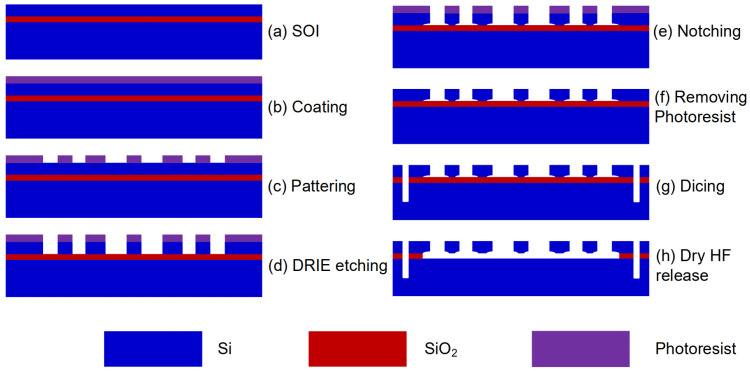
SOI fabrication process of the device.

Using a standard silicon-on-insulator (SOI) process as depicted in Figure 4, the device was fabricated. The thickness of the device layer, handle layer, and oxide layer was 60, 400, and 4 μm, respectively (Figure 4a). The fabrication process shown in Figure 4 contains photoresist spin coating (b), patterning (c), DRIE etching (d), notching (e), removing photoresist (f), dicing (g), and dry HF release structure. The basic parameters of the fabricated accelerometer (Figure 3) are measured and listed in Table 2. In addition, the mechanical parameters and close-up view of the fabricated device are given in the Supporting Information as an attachment for the readability of this paper.

The developed accelerometer was packaged in a vacuum of 1 bar and tested under ambient pressure at room temperature. Mounted on the dividing head, the sensor responded to the component of gravity. The output of the sensor was sampled by the DAQ [34] at a rate of 2.5 MHz, and the responding time clock resolution (reciprocal of sampling rate) was 4 × 10^−7^ s. And DRPs were set as *X*_1_ = 0 nm and *X*_2_ = 130 nm because they are optimal for this developed sensor [31]. These parameters were used throughout the experiment. Then, time intervals were measured, and sensitivity and adjustment mechanisms were analyzed.

## 4. Results and Discussion

### 4.1. Sensitivity Measurement and Comparison of Measured Nonlinearity

**Figure 5:**
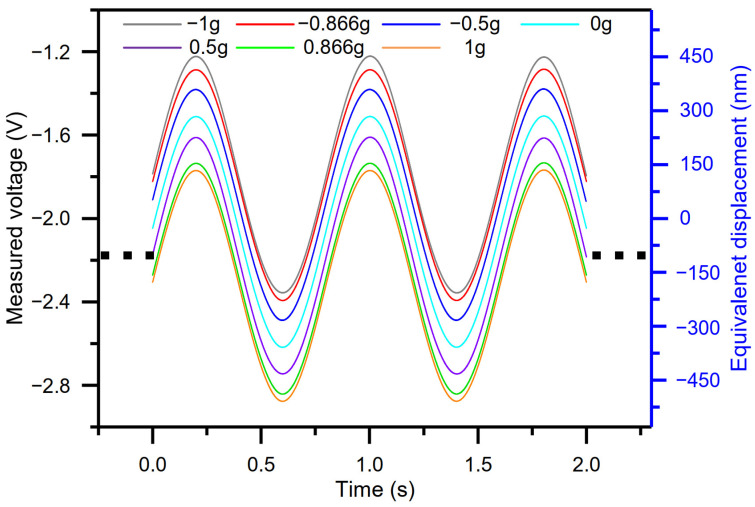
Sampled oscillation trajectories of different components of gravitational force (the blank dot denotes that the oscillation trajectories display periodic repetitiveness, whereas part of the oscillation trajectories is captured and drawn).

The accelerometer works under a resonant state. With reference to the relationship between equivalent displacement and output voltage [31], the vibration amplitudes are driven and adjusted to near ~342 nm. Figure 5 shows the sampled oscillation trajectory concerning different components of gravitational force. According to the time interval measurement method [31] and the sampled data, time intervals are measured, and acceleration is solved in different acceleration perturbations (Table 3) on the condition that resonant frequency is 1245.88 Hz; DRP *X*_1_ is 0 nm and DRP *X*_2_ is 130 nm. As can be seen from Table 3, the measured time intervals Δ*T*_1_ and Δ*T*_2_ vary with the different acceleration perturbations while the measured time interval Δ*T* is almost constant, which indicates that these experimental results coincide well with the theoretical analysis. The minimum value and maximum value of Δ*T* are 802.4 μs and 802.8 μs, respectively. The difference of 0.4 μs is caused by sensor noise. In this work, as the signal is sampled at a rate of 2.5 MHz, the time clock resolution is 0.4 μs, and the difference between measured time intervals Δ*T* is also 0.4 μs.

**Figure 6:**
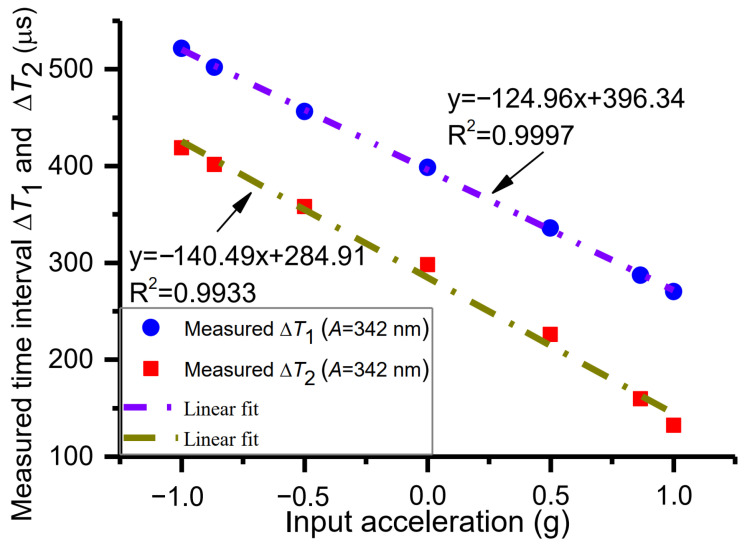
Measured sensitivities *S*_1_ and *S*_2_ and nonlinearity.

As presented in Figure 6, the measured sensitivity *S*_1_ is −124.96 μs/g in the range of −1 g to +1 g. The linear fitting function between the time interval Δ*T*_1_ and acceleration is y=−124.36x+396.34 with *R*^2^ = 0.9997, while the measured sensitivity *S*_2_ is −140.49 μs/g in the range of −1 g to +1 g. The linear fitting function between the time interval Δ*T*_2_ and acceleration is y=−140.49x+284.91 with *R*^2^ = 0.9933. Thus, the nonlinearity of the sensitivity *S*_1_ is smaller than that of the sensitivity *S*_2_. Beyond that, the measured result of nonlinearity is consistent with the theoretical analysis and the simulated result. As a result, the sensitivity *S*_1_ is preferred for evaluating time domain sensors.

### 4.2. Measurement and Discussion of Adjustable Sensitivity Resulting from Varying Vibration Amplitude

**Figure 7:**
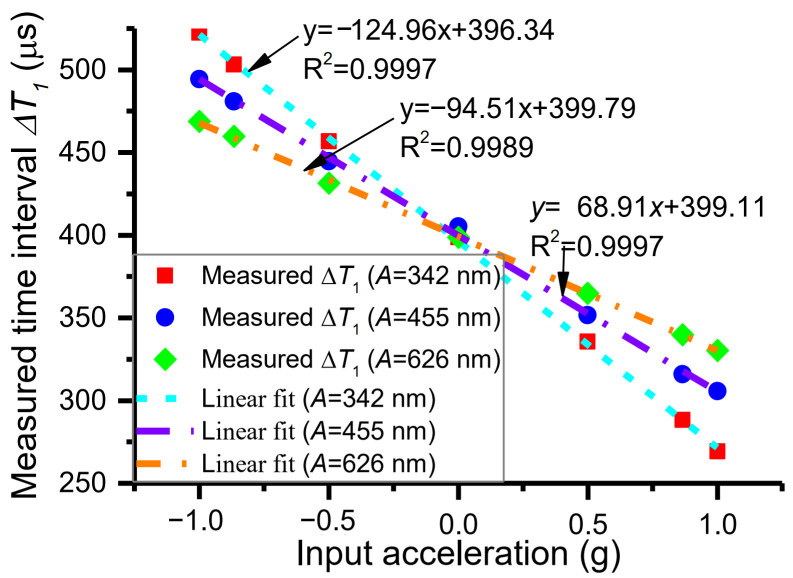
Measured sensitivities of different vibration amplitudes.

**Figure 8:**
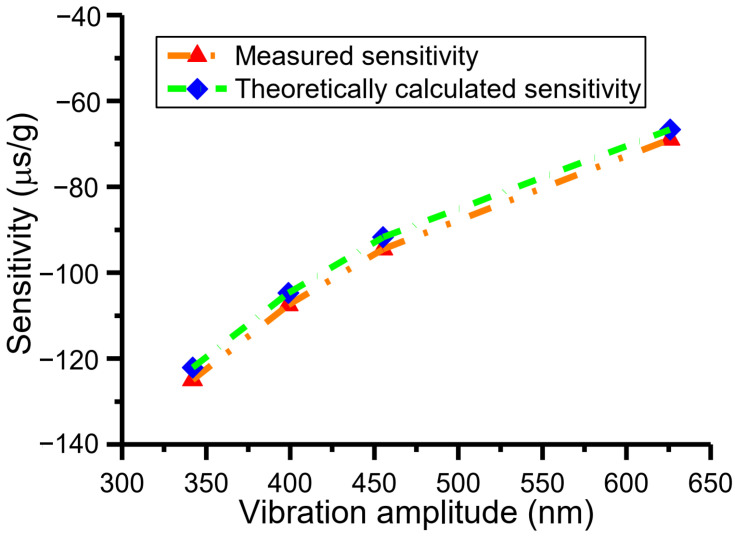
Comparisons of the measured and theoretically calculated sensitivities at different vibration amplitudes.

The time intervals, Δ*T*_1_, Δ*T*_2_, and Δ*T*, and acceleration are measured under vibration amplitudes of 455 nm and 626 nm, respectively, with the same method as above. The vibration amplitudes are adjusted by changing the electrostatic driving force. The dependence of measured time intervals Δ*T*_1_ on acceleration perturbation under different vibration amplitudes is shown in Figure 7. As can be observed from Figure 7, when the vibration amplitudes are 342 nm, 455 nm, and 626 nm, the measured sensitivities are −124.96 μs/g, −94.51 μs/g, and −68.91 μs/g, respectively, on the condition that the resonant frequency is 1246.26 Hz and DRP *X*_1_ is 0 nm. At the same time, the theoretical sensitivities are −122.09 μs/g, −91.73 μs/g, and −66.70 μs/g, respectively. The comparisons of measured and theoretical sensitivities at different amplitudes are shown in Figure 8, which indicates that the experiment is consistent with the theory. As can be seen from Figure 8, the measured sensitivities are slightly larger than theoretically calculated sensitivities. The reasons are shown below. The resonant frequency for the theoretical sensitivity is extracted from the parameters of the accelerometer, and it is 1245.88 Hz (Table 2). The resonant frequency for the measured sensitivity is derived through the measured time interval Δ*T*; i.e., the resonant frequency is the reciprocal of the measured time interval Δ*T*. The magnitude of the resonant frequency is about 1245.42 Hz. Due to the sensitivity being inversely proportional to the third power of resonant frequency, the measured sensitivity is slightly larger than the theoretically calculated sensitivity. Notably, the vibration amplitude cannot be adjusted arbitrarily. The interrelationships among vibration amplitude, measurement range, and DRPs were presented in [31].

Compared with amplitude, the resonant frequency of the time domain sensor has a greater influence on sensitivity (Section 2.3). Unfortunately, in this work, the resonant frequency of the sensor could not be varied because the tuning electrode was not arranged. A device with a special port for tuning resonant frequency will be designed for a sensitivity adjustment test in the next work.

### 4.3. Measurement and Analysis of Tunable Sensor Performance Based on Adjustable Vibration Parameters or Sensitivities

**Figure 9:**
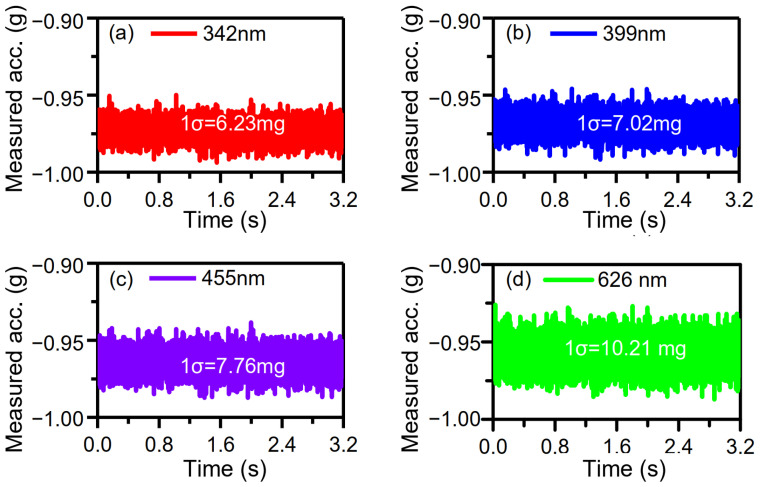
Dependence of the measured acceleration on different vibration amplitudes: (**a**) 342 nm, (**b**) 399 nm, (**c**) 455 nm, (**d**) 626 nm.

**Figure 10:**
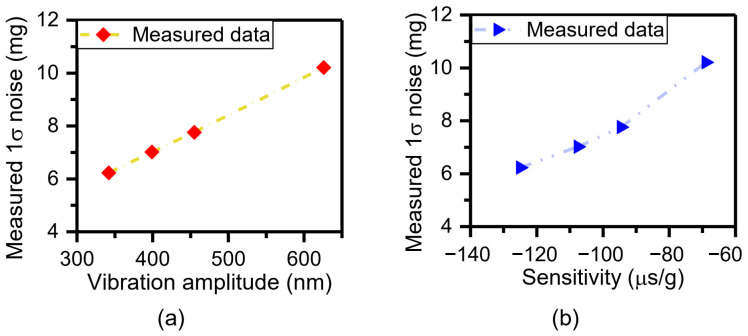
Dependence of the 1σ noise on adjustable vibration amplitudes (**a**) and sensitivities (**b**).

The performance of the time domain accelerometer can be evaluated by the standard deviation (1σ) of the measured acceleration values [31]. The output of the sensor is sampled at the maximum input acceleration of −1 g under different vibration amplitudes. The time length of the sampled signal is 3.2 s, and the sampling rate is 2.5 MHz. Then, DRP *X*_1_ is 0 nm, and DRP *X*_2_ is 130 nm. Finally, the solved acceleration is depicted in Figure 9. The 1σ noises of solved acceleration are 6.23 mg, 7.02 mg, 7.76 mg, and 10.21 mg at the different vibration amplitudes of ~342 nm, ~399 nm, ~455 nm, and ~626 nm, respectively. Therefore, the relationship between the 1σ noises and vibration amplitudes is shown in Figure 10a, which indicates that a smaller vibration amplitude corresponds to a smaller 1σ noise. In addition to that, it was already verified that the sensitivity can be adjusted by varying vibration amplitude (Section 4.2). The measured sensitivities are −124.96 μs/g, −107.54 μs/g, −94.51 μs/g, and −68.91 μs/g at the variable vibration amplitudes of ~342 nm, ~399 nm, ~455 nm, and ~626 nm (Figure 8). Therefore, the dependence of the 1σ noises on sensitivity is shown in Figure 10b, which depicts that the larger the sensitivity, the smaller the 1σ noise. This feature of the time domain sensor is consistent with that of a variety of sensors, such as a magnetometer for high-resolution magnetic resonance spectroscopy [35], biosensors [36], electrochemical sensors [37], and displacement sensors [38]. In other words, sensor noise can be lowered by improving the sensor sensitivity. Consequently, there can be a conclusion for time domain sensors that the bigger the vibration, the smaller the sensitivity and the bigger the noise. Moreover, the adjustable sensitivity provides an opportunity to build a range self-adaptive time domain sensor. It should be noted that for the developed time domain sensor of this paper, displacement of the proof mass is indirectly represented by the output voltage of the capacitance device. The noise floor is determined by not only capacitance-to-voltage (C–V) interface circuit noise and Brownian noise, but also the measurement accuracy of time. The acceleration resolution caused by the time clock resolution equals the time measurement accuracy divided by sensitivity. Therefore, the time clock resolution of 4 × 10^−7^ s (far less than the state-of-the-art time accuracy of 10^−18^ s) corresponds to an acceleration resolution of 3.20 mg at the sensor amplitude of ~342 nm. In addition to that, the acceleration resolution of 3.20 mg is introduced to the developed sensor and is more than half of the 1σ noise of 6.23 mg. Moreover, the measured acceleration resolution of the capacitance device is 2.52 mg, mainly caused by the C–V interface circuit. [31]. As a result, the time measurement accuracy and the C–V interface circuit are the two main causes of the noise floor. Furthermore, the accuracy of the developed accelerometer is lower than that of the capacitive or resonant accelerometers built with the same device and the interface circuit because of the introduced time measurement. If the displacement and DRPs of the device are physically defined as stacked tunneling electrodes [24], the C–V interface circuit is not necessary at all for defining the displacement and DRPs. Thus, there exists no noise from the C–V interface circuit and Brownian noise coupled to the output [24]. The noise floor is only determined by the time measurement accuracy. Beyond that, time measurement accuracy is the highest in the international system of units. Thus, the time domain accelerometer that converts acceleration into time has promising prospects in high accuracy.

Compared with vibration amplitude, resonant frequency has a larger influence on sensitivity (Section 2.3). Apart from that, utilizing the measured result that the sensor performance can be tuned by its sensitivity, the sensor performance can be significantly tuned through the resonant frequency. Due to the sensor of this work being built without frequency-tuning electrodes, a sensor with a frequency-tuning electrode will be developed for sensor noise testing. In addition, the sensor noise caused by time clock resolution will be theoretically analyzed and experimentally verified.

## 5. Conclusions

A characterization of the sensitivity of time domain sensors has been performed. The sensitivity can be adjusted via vibration amplitude and resonant frequency. Resonant frequency has a relatively larger impact on it compared with vibration amplitude. The experimental results show that the designed time domain accelerometer has different sensitivity from −68.91 μs/g to −124.96 μs/g (amplitude of 626 nm: −68.91 μs/g; amplitude of 455 nm: −94.51 μs/g; amplitude of 342 nm: −124.96 μs/g). In addition, the performance of time domain sensors can be improved by adjustable sensitivity (−68.91 μs/g: 10.21 mg; −94.51 μs/g: 7.76 mg; −124.96 μs/g: 6.23 mg). The adjustable sensitivity provides a possibility for range self-adaption of the time domain sensor. Due to the limitations of the current device and test platform, the resonant frequency cannot be tuned. Thus, a sensitivity adjustment test with varying resonant frequency has not been conducted. A device with a special port for tuning resonant frequency will be designed for sensitivity enhancement and adjustment tests in future work. As another special port is used for loading AC electric signals (equivalent to AC acceleration), it will be also designed and added to the device for range self-adaptive tests. The results reported in this paper can be also applied to other time domain inertial sensors, e.g., a time domain gyroscope or tilt sensor.

## Figures and Tables

**Table 1:** Comparison of the time domain accelerometer with three traditional transducer-based accelerometers.

	Capacitive and Similar [3,8,9,10,11]	Resonant [4]	Mode- Localized [5]	Time Domain [24,31]
Proof mass	Quasi-static	Quasi-static	Quasi-static	Resonant
Elastic beam	Quasi-static	Resonant	Resonant	Quasi-static
Converting acceleration into	Displacement	Frequency	Amplitude ratio	Time intervals
Measured quantity for representing acceleration	Voltage or current	Voltage or current	Voltage or current	Time intervals
Mechanical sensitivity	m/g	Hz/g or ppm/g	1/g or ppm/g	s/g
Interface circuit gain	V/m or A/m	1 *	1 *	1 *
Total sensitivity **	V/g or A/g	Hz/g	ppm/g	s/g

* The interface circuit converts mechanical vibration into an electrical signal that is easily measured so that the interface circuit gain is 1. ** The total sensitivity equals mechanical sensitivity multiplied by interface circuit gain.

**Table 2:** Accelerometer parameters *.

Parameters	Value
Resonant frequency	1245.88 Hz
Measurement range	±1 g
Bandwidth	1.25 Hz
Quality factor	~1000

* Values of parameters are from [31].

**Table 3:** Measured time intervals and acceleration under different acceleration perturbations.

Input Acceleration (g)	Measured Time Intervals (μs)			Measured Acceleration (g)
	Δ*T*_1_	Δ*T*_2_	Δ*T*	
−1	521.6	418.8	802.4	−0.96335
−0.866	502.0	401.6	802.4	−0.81670
−0.5	456.4	358.0	802.4	−0.45568
0	398.4	298.0	802.4	0.02333
0.5	336.0	226.0	802.4	0.53922
0.866	287.2	159.6	802.8	0.92645
1	270.4	132.4	802.8	1.05361

## Data Availability

Data are contained within the article.

## Supplementary Materials

The following supporting information can be downloaded at https://www.mdpi.com/article/10.3390/mi15020227/s1, Figure S1: Lumped parameter model of time domain accelerometer; Figure S2: Oscillation trajectory of the proof mass versus time with (blue line) and without (red line) external AC acceleration (a) and the DRPs, trigger events and extracted time intervals in one period of oscillation (b); Figure S3: SEM image and close-up of the time domain accelerometer; Table S1: Mechanical parameters of the fabricated device.
