# Supplementary material for: Characterization of Sensitivity of Time Domain MEMS Accelerometer
Source: Micromachines (Basel). 2024 Jan 31;15(2):227. doi: 10.3390/mi15020227 (PMC10891993; doi:10.3390/mi15020227)
Supplement: Supplementary file 1 [file micromachines-15-00227-s001.zip › micromachines-2830843-supplementary.pdf]

Supporting Information

Time domain acceleration sensing mechanism

The time domain acceleration sensing mechanism is described in reference [1] and Appendix A. Supplementary data [2]. In this supporting information, some significant contents and parameters appearing in the manuscript are outlined from the literature. Noticeably, all the information of this supporting information comes from the reference [1] and [2]. Moreover, ΔT_1, ΔT_2, and ΔT denote time intervals in the manuscript while T_1, T_2, and T denote time intervals in the literature. Other related parameters remain the same as the literature.

As shown in Figure. S1, the model of the time domain accelerometer is equivalent to a forced vibration of a mass–spring–damper single freedom system. Therefore, the motion equation of the proof mass can be expressed by

$$m\frac{d^2x}{dt^2}+c\frac{dx}{dt}+\ kx=-m\cdot a\sin(\omega_a t)+H\sin(\omega t).$$ ◎S1◎◎

where m denotes the mass, x denotes the displacement of the proof mass related to rest position, c denotes the viscous damping coefficient, k denotes the spring coefficient, a and ω_a denote the amplitude and frequency of the external alternating current (AC) acceleration applied to the proof mass, respectively, and H and ω denote the amplitude and frequency of the harmonic excitation force F, respectively. The displacement of the proof mass with external acceleration in steady-state (Figure. S2(a)) is given by

$$x=A\cos(\omega_0 t)-C\sin(\omega_a t-\varphi)$$ ◎S2◎

where A is vibration amplitude of harmonic oscillation, and $\omega_0^2=k/m=(2\pi f_0)^2$. $A\cos(\omega_0 t)$ is oscillation displacement of the proof mass without external acceleration (Fig. S2(a)). $C\sin(\omega_a t-\varphi)$ is the displacement caused by the external AC inertial force, i.e., the center position of the oscillation trajectory with external acceleration is shifted from that of a zero-acceleration oscillation trajectory as shown in Figure. S2(a).

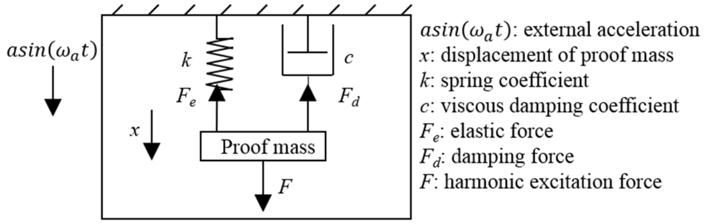

Figure. S1. Lumped parameter model of time domain accelerometer.

As shown in Figure. S2, in order to convert the measurement of this displacement into that of time, two displacement reference points (DRPs), X_1 and X_2, are defined. When the proof mass with external inertial force passes the first displacement reference point of $x = X_1$, three trigger events tr_1, tr_4, and tr_5 (marked as black circles) are successively generated in the period of

oscillation; the times of these three trigger events is t_1, t_4, and t_5, respectively. The period of oscillation is defined from t_1 to t_5. Time interval ΔT_1 is determined by subtracting the times of trigger events tr_1 and tr_4, i.e., $\Delta T_1 = t_4 - t_1$. Time interval ΔT is determined by subtracting the times of trigger events tr_1 and tr_5, i.e., $\Delta T = t_5 - t_1$. In the same way, when the proof mass with external inertial force passes the second displacement reference point of x = X_2, two trigger events tr_2 and tr_3 (marked as black triangles) are successively generated in the period of oscillation. The time of the two trigger events tr_2 and tr_3 is t_2 and t_3, respectively. The time interval ΔT_2 is determined by subtracting the times of successive trigger events tr_2 and tr_3, i.e., $\Delta T_2 = t_3 - t_2$. The extracted time interval ΔT is the period of oscillation at the time. The frequency ω_0 can be expressed by the extracted parameter ΔT and constant 2π, i.e., $\omega_0 = 2\pi/\Delta T$. Finally, the acceleration can be solved by the measured time intervals by

$$a = \left(\frac{2\pi}{T}\right)^2 \left(\frac{(X_1-X_2)\cos\left(\pi\frac{\Delta T_1}{\Delta T}\right)}{\cos\left(\pi\frac{\Delta T_1}{\Delta T}\right)-\cos\left(\pi\frac{\Delta T_2}{\Delta T}\right)} - X_1\right). \qquad \text{(S3)}$$

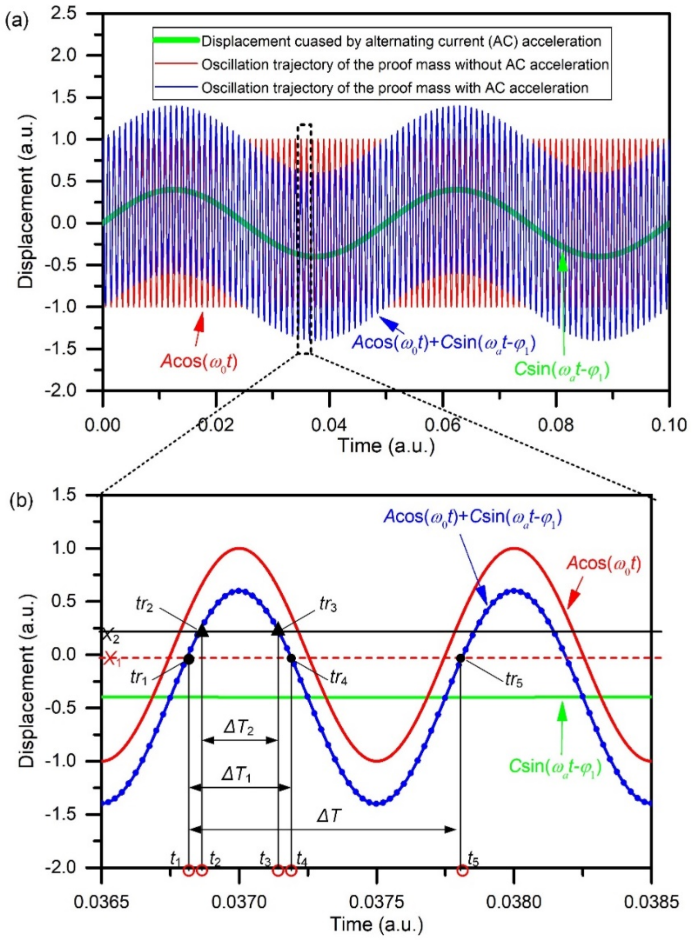

Figure. S2. Oscillation trajectory of the proof mass versus time with (blue line) and without (red line) external AC acceleration (a) and the DRPs, trigger events and extracted time intervals in one period of oscillation (b).

In this paper, the displacement of the proof mass is indirectly represented by the output voltage of the capacitance device while DRPs are represented by the voltage reference points (VRPs). The representation is implemented by the capacitance device and capacitance-to-voltage (C–V) interface circuit. The relationship between displacement and voltage is deduced as follow. The sensor's sensing axis is directly pointed at the Earth's center. The direct current (DC) voltage of +1g reading (V_{1g}) is extracted. Similarly, the sensor is turned 180° to measure −1g and the DC voltage of −1g reading (V_{-1g}) is extracted. Hence, the sensitivity of displacement to voltage S_{xV} can be obtained by

$$S_{xV} = \frac{2g/\omega_0^2}{V_{1g}-V_{-1g}} \quad \text{(S4)}$$

Then the relationship between displacement x and voltage V can be expressed by

$$x = \left(V - \frac{V_{1g}+V_{-1g}}{2}\right) \times S_{xV} \quad \text{(S5)}$$

When we set $x = X_1$ according to Equation. (S5), the corresponding voltage reference point V_1 is obtained. In the same way, the voltage reference point V_2, which corresponds to displacement reference point X_1, is also obtained.

Details information of the developed device

The time domain accelerometer was described in reference [1] and [2]. In this supporting information, some significant figures and contents as well as parameters appearing in the manuscript are outlined from the literatures. Noticeably, all the information of this supporting information comes from the reference [1] and [2].

Figure S3 shows the SEM image and close-up of the time domain accelerometer.

Table S1 shows the developed accelerometer parameters.

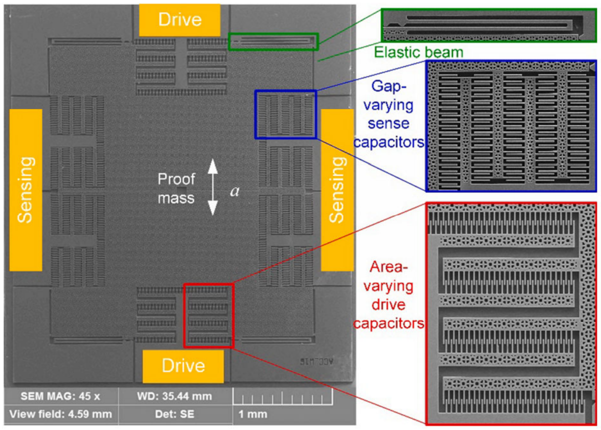

Figure. S3. SEM image and close-up of the time domain accelerometer.

Table S1. Mechanical parameters of the fabricated device.

Parameters	Value
Thickness of the device layer	30 μm
Overall sensor size	3.5 mm × 3.8 mm
Proof mass	2.4×10^{-7} kg
Capacitance gap	2.5 μm
Driving capacitance	1 pF
Sensing capacitance	3.5 pF
Capacitance sensitivity	0.2 pF/g
Spring stiffness	15 N/m
Designed resonant frequency	1250 Hz
Quality factor	~1000
Mechanical noise	70 ng/√Hz

Reference

[1] E. Li, Q. Shen, Y. Hao, and H. Chang, "A Virtual Accelerometer Array Using One Device Based on Time Domain Measurement," IEEE Sens. J., vol. 19, no. 15, pp. 6067–6075, 2019.

[2] E. Li; L. Zhang; X. Guan; et al., "Novel acceleration measurement method during attenuation vibration of inertial sensor based on time domain sensing mechanism," Measurement, 218(2023): 113077.
